# Editorial: RNA-binding proteins in cancer: advances in translational research

**DOI:** 10.3389/fcell.2024.1390044

**Published:** 2024-03-08

**Authors:** Caterina Mancarella, Nadine Bley, Luiz O. F. Penalva

**Affiliations:** ^1^ Laboratory of Experimental Oncology, IRCCS Istituto Ortopedico Rizzoli, Bologna, Italy; ^2^ Institute of Molecular Medicine, Martin Luther University Halle-Wittenberg, Halle, Germany; ^3^ Greehey Children’s Cancer Research Institute, University of Texas Health Science Center at San Antonio, San Antonio, TX, United States; ^4^ Department of Cell Systems and Anatomy, University of Texas Health Science Center at San Antonio, San Antonio, TX, United States

**Keywords:** RNA-binding proteins, cancer targets, prognosis biomarkers, post-transcriptional mechanisms, network analysis

RNA-binding proteins (RBPs) regulate RNA metabolism, processing, and translation by establishing highly dynamic interactions with hundreds of coding and non-coding target RNAs within ribonucleoprotein complexes (Van Nostrand et al., 2020). Alterations in RBP expression levels or function contribute to several pathological conditions, including cancer. Though underappreciated in the past, RBPs are now recognized as a new class of cancer drivers (Gebauer et al., 2021). Most cancer hallmarks, including immune system evasion, stemness, proliferation, and cell dissemination, are regulated by RBPs (Zhou et al., 2023). Thus, the RBP contribution to cancer phenotypes can be explored in alternative therapeutic strategies.

This Research Topic embraced the most recent translational findings in RBP-mediated processes in cancer. As Editors, we had the pleasure to accept for publication five original research articles and one review, which discussed RBPs’ role in post-transcriptional processes and their implications for tumor subtyping, response to treatment, and survival.

As highlighted by García-Cárdenas et al., the identification of tumorigenic RBPs could fulfil the need to discover accurate and sensitive therapeutic targets for specific tumor subtypes. The authors highlighted that distinct RBPs are involved in colon (COAD) and rectal (READ) carcinomas. By combining genomic, transcriptomic, proteomic, and interactions data from 488 COAD, 155 READ patients, and 102 cancer cell lines, they were able to assign oncogenic RBPs for each tumour subtype. In COAD, the authors identified NAT10, NOP56, RBM12 and FKBP1A while in READ, CSE1L, and EMG1 were pointed out as the ones with the strongest potential for clinical applications.

Among tumor-specific studies, Zhang et al. focused on RBM24, an RBP mechanistically involved in muscle cell differentiation and lens development. The authors demonstrated that RBM24-deficiency led to increased liver steatosis and chronic inflammation but not spontaneous tumors in mice. RBM24 protects cancer cells from ferroptosis, a type of programmed cell death characterized by lipid reactive oxygen species accumulation which induces plasma membrane damage. Mechanistically, RBM24 acts by stabilizing *SLC7A11* mRNA, an inhibitor of ferroptosis. This study reveals a novel post-transcriptional function of RBM24 as regulator of lipid metabolism, and highlights for the first time a specific role for RBM24 in cancer.

In their review, Montiel-Dávalos et al. discussed the relationship between translation initiation and the translation machinery in glioblastoma. Among the crucial regulators of RNA translation, the authors discussed the role of specific RBPs in hypoxia response. In particular, a network of hypoxia-sensitive RBPs, including HuR, PCBP1, RBM4, and hnRNP A2/B1, represents a new complex that functions as a translation activation switch of anaerobic metabolism (Ho et al., 2020) with relevant implications in hypoxia response in glioblastoma cells.

Three articles in this Research Topic focused on the construction of signatures of cancer risk based on specific RNA epigenetic modifications. RNA epigenetic refers to specific modifications of RNA, which profoundly affect RNA destiny and cellular behaviour. RNA modifications include N6-methyladenosine (m6A), N7-methylguanosine (m7G), and 5-methylcytidine (m5C) (Tang et al., 2023). He et al. performed a pan-cancer analysis focusing on the m7G modification, which is widespread in numerous RNA cap structures as well as in tRNA, rRNA, mRNA and miRNA. m7G affects all the stages of RNA processing including splicing, export, decay, and controls the mRNA translation. The authors considered the m7G-related regulators in pan-cancer datasets, and further evaluated the correlation of the differentially expressed m7G-related regulators with patient survival. The authors identified a signature composed of *WDR4, METTTL1, NUDT1, CYFIP2,* and the *RBP IFIT5*, associated with poor prognosis in cancer, including carcinoma and sarcoma patients.

Liu et al. focused on m6A modification which is overall governed by demethylases (“eraser”), methyltransferases (“writer”), and RBPs (“reader”). Several studies have demonstrated that genetic alterations or dysregulated expression of m6A regulators play a role in the development, advancement, and treatment response of cancers. The authors investigated the expression patterns and prognostic value of 17 m6A regulators in 551 prostate cancer samples to construct a comprehensive diagnostic scoring model and to create a complete atlas of prognosis-related m6A regulators. The authors found that the expression levels of RBM15, METTL3, YTHDC2, YTHDF1/2, HNRNPC, and HNRNPA2B1 in samples were upregulated compared to normal samples. Of those, 3 m6A regulators, including METTL14 and the RBPs HNRNPA2B1, and YTHDF2 exhibited relatively higher prediction accuracy than PSA. In addition, univariate and multivariate analyses confirmed a strong correlation between this “3-genes risk scores” and clinicopathological features of prostate cancer.

Yun et al. investigated the prognostic value of tumor-associated dysregulation of the m5C-modification pathway for pancreas cancer (PDAC) patients. m5C-methylation regulators include ten writers, one eraser and one reader. Of those, by combining genomic, transcriptional, and clinical data from 239 PDAC patients, the authors identified three genes (*DNMT1*, *NSUN4*, and the RBP *YBX1*) as upregulated in tumors compared to normal counterparts. High *YBX1* expression was associated with sensitivity to the PI3Kβ inhibitor AZD6482, and EGFR inhibitor Gefitinib. In addition, *YBX1* expression was associated with an active anti-tumor immune response, demonstrating the potential wide impact of this RBP in PDAC.

**FIGURE 1:**
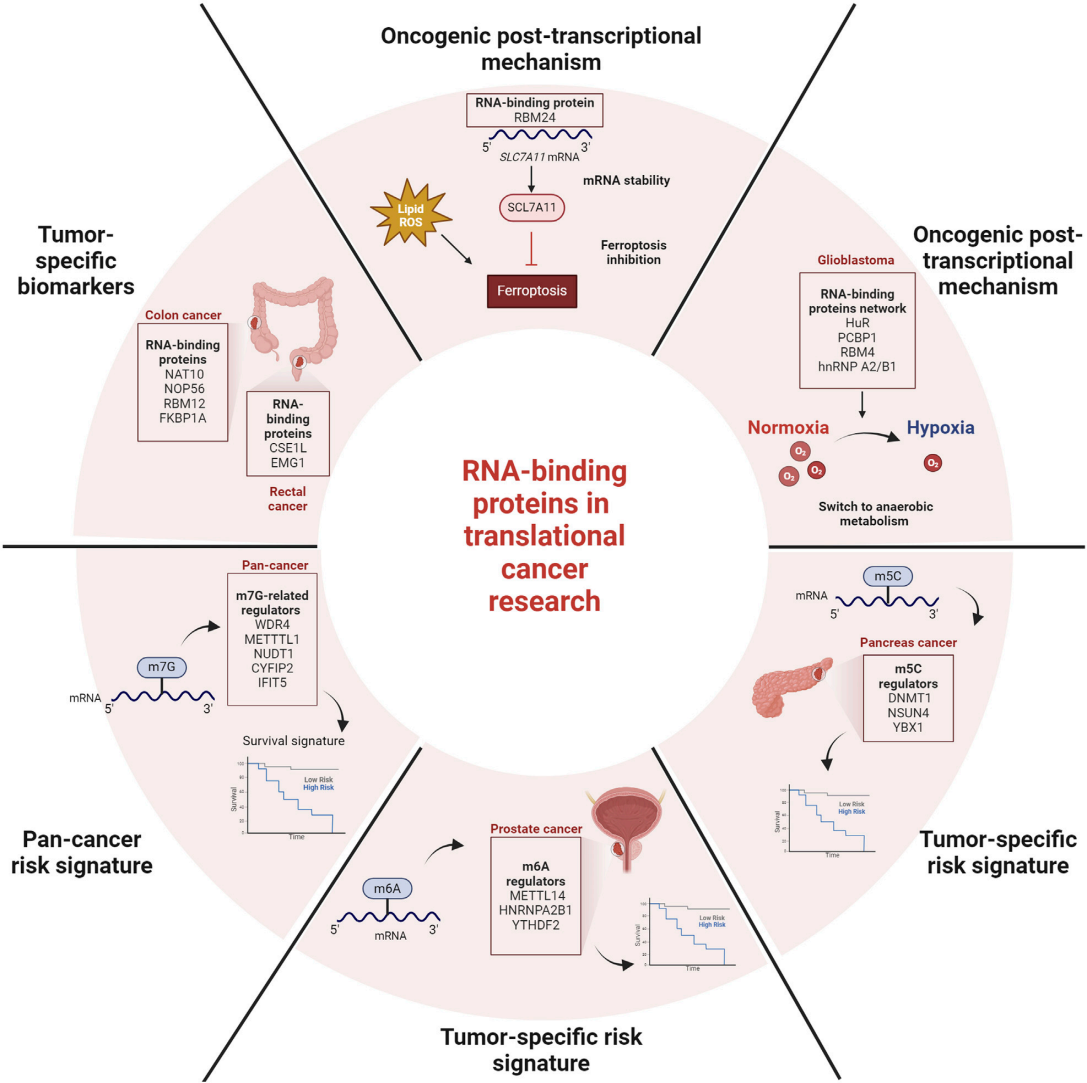
Summary of discussed RBP-related post-transcriptional processes and implications for deciphering cancer aggressiveness and patients subtyping.

In conclusion, the information obtained to date indicates that RBPs play a role in cancer development and progression. However, the specific roles of the majority of these RBPs, including biological functions and mechanisms, remain unknown. Additionally, some of them exhibit pleiotropic and complex actions, including the recognition of specific epigenetic RNA modification, opening a new frontier of research. As summarized in Figure 1, the information collected in this Research Topic supports the evaluation of RBPs and their interactors to unveil novel oncogenic mechanisms and identify novel biomarkers. Although further investigations are needed, RBPs have the potential to become powerful tools in the fight against cancer.
